# Choline Kinase Alpha Inhibition by EB-3D Triggers Cellular Senescence, Reduces Tumor Growth and Metastatic Dissemination in Breast Cancer

**DOI:** 10.3390/cancers10100391

**Published:** 2018-10-22

**Authors:** Elena Mariotto, Giampietro Viola, Roberto Ronca, Luca Persano, Sanja Aveic, Zaver M. Bhujwalla, Noriko Mori, Benedetta Accordi, Valentina Serafin, Luisa Carlota López-Cara, Roberta Bortolozzi

**Affiliations:** 1 Pediatric Hematooncology Laboratory, Department of Women’s and Children’s health, University of Padova, 35128 Padova, Italy; luca.persano@unipd.it (L.P.); benedetta.accordi@unipd.it (B.A.); valentina.serafin@unipd.it (V.S.); roberta.bortolozzi@unipd.it (R.B.); 2 Fondazione Istituto di Ricerca Pediatrica Città della Speranza, 35127 Padova, Italy; s.aveic@irpcds.org; 3 Experimental Oncology and Immunology, Department of Molecular and Translational Medicine, University of Brescia, 25123 Brescia, Italy; roberto.ronca@unibs.it; 4 In Vivo Cellular Molecular Imaging Center Program, Russell H. Morgan Department of Radiology and Radiological Science, John Hopkins University, Baltimore, MD 21205, USA; zaver@mri.jhu.edu (Z.M.B.); noriko@mri.jhu.edu (N.M.); 5 Department of Pharmaceutical Chemistry and Organic, Faculty of Pharmacy, Cartuja Campus, University of Granada, 18011 Granada, Spain; lcarlotalopez@ugr.es

**Keywords:** Choline Kinase α, breast cancer, small molecules, senescence

## Abstract

Choline kinase (ChoK) is the first enzyme of the Kennedy pathway leading to the biosynthesis of phosphatidylcholine (PtdCho), the most abundant phospholipid in eukaryotic cell membranes. EB-3D is a novel choline kinase α1 (ChoKα1) inhibitor with potent antiproliferative activity against a panel of several cancer cell lines. ChoKα1 is particularly overexpressed and hyperactivated in aggressive breast cancer. By NMR analysis, we demonstrated that EB-3D is able to reduce the synthesis of phosphocholine, and using flow cytometry, immunoblotting, and q-RT-PCR as well as proliferation and invasion assays, we proved that EB-3D strongly impairs breast cancer cell proliferation, migration, and invasion. EB-3D induces senescence in breast cancer cell lines through the activation of the metabolic sensor AMPK and the subsequent dephosphorylation of mTORC1 downstream targets, such as p70S6K, S6 ribosomal protein, and 4E-BP1. Moreover, EB-3D strongly synergizes with drugs commonly used for breast cancer treatment. The antitumorigenic potential of EB-3D was evaluated in vivo in the syngeneic orthotopic E0771 mouse model of breast cancer, where it induces a significant reduction of the tumor mass at low doses. In addition, EB-3D showed an antimetastatic effect in experimental and spontaneous metastasis models. Altogether, our results indicate that EB-3D could be a promising new anticancer agent to improve aggressive breast cancer treatment protocols.

## 1. Introduction

Metabolic reprogramming has been recognized as one of the 10 hallmarks of cancer [1]. Malignant cells need to change their cellular energy metabolism to support unrestrained cell proliferation and to adapt to new microenvironmental conditions and to different challenges. Lipid metabolism is no exception: a sustained biosynthesis of membrane phospholipids is required to meet the demand of rapidly proliferating cells. In fact, alteration in choline (Cho) metabolism has been observed in many cancers [2,3,4,5,6,7,8] and it has been related to deregulated cell proliferation, invasion, and metastasis. The so-called “cholinic phenotype” consists of increased level of phosphocholine (PCho) and, in general, of total choline-containing metabolites (tCho) mainly due to the overexpression and/or hyperactivation of the α1 isoform of choline kinase (ChoKα1) [9,10]. In humans, three isoforms of ChoK have been described: ChoKα1 and ChoKα2 encoded by the *CHKA* gene and ChoKβ by *CHKB.* In the first step of the Kennedy pathway, these enzymes catalyze the phosphorylation of choline to phosphocholine, ultimately leading to the synthesis of phosphatidylcholine (PtdCho), the most abundant phospholipid of the eukaryotic cell membrane. Although ChoK proteins share high sequence similarity, only the ChoKα1 isoform has been proposed as an oncogenic promoting factor. Increased expression of ChoKα1 has been extensively described in breast cancer, where significantly increased activity has been reported in 40% of patients in correlation with histological tumor grade and poor clinical outcome [5]. Silencing of *CHKA* by RNA interference has been demonstrated to reduce cell proliferation and tumor growth [11,12], sensitize cancer cells to chemotherapeutics [13], and suppress migration and invasion, while *CHKB* silencing has no effect [3,9]. For these reasons, ChoKα1 has been proposed as a new appealing target for cancer therapy, and during the past decades, extensive efforts have been made to synthesize and improve ChoKα1 inhibitors [14,15,16,17,18,19,20,21,22,23].

EB-3D (previously named 10a) is a novel choline-competitive symmetrical biscationic ChoKα inhibitor that was shown to impair cell proliferation in a panel of different cancer cell lines [24]. Our group recently reported that EB-3D is able to induce apoptosis in T cell acute lymphoblastic leukemia and to synergize with L-asparaginase by targeting the same signaling pathway [8]. Here, we further investigated the effect of EB-3D-mediated ChoKα inhibition both in vitro and in vivo in breast cancer, where the overexpression and hyperactivation of ChoKα are associated with tumor progression, invasion, and metastasis [13,25]. We found that EB-3D, through phosphocholine level reduction, was able to impair cell proliferation, thus triggering cells to senescence via activation of the AMPK-mTOR pathway. Moreover, EB-3D treatment significantly enhanced the antitumorigenic potential of drugs commonly used in breast cancer treatment protocols. Finally, the inhibition of ChoKα by EB-3D reduced cell invasion and migration, with a significant loss of metastatic potential of breast cancer cells in vivo.

## 2. Results

### 2.1. Inhibition of ChoKα by EB-3D Reduces Soluble Choline Metabolites

**Figure 1:**
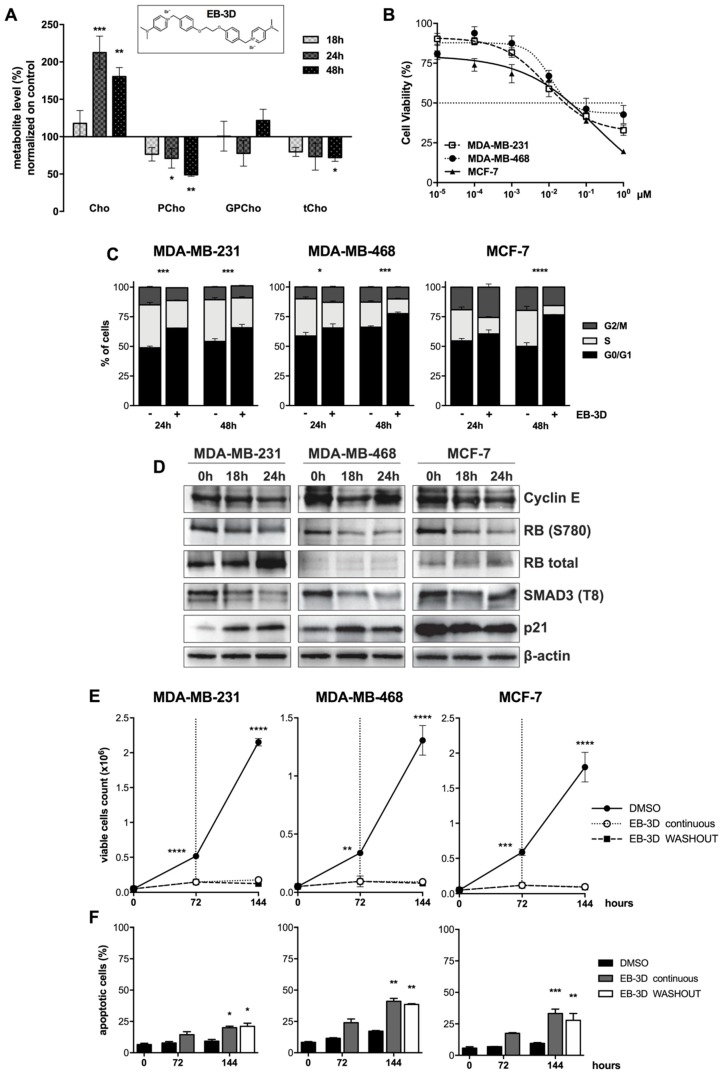
Effect of EB-3D choline kinase α (ChoKα) inhibition in breast cancer cells. (**A**) Levels of choline (Cho), phosphocholine (PCho), glycerophosphocholine (GPCho), and total choline-containing compounds (tCho) quantified from 1H-NMR spectra of water-soluble extracts from MDA-MB-231 cells treated with DMSO or 1 μM of EB-3D (chemical structure shown in the inset) for the indicated time points. Metabolite levels are expressed as a percentage with respect to the control. (**B**) MTT cell viability assay in MDA-MB-231, MDA-MB-468 and MCF-7 cell lines treated with EB-3D for 72 h. The percentages of cell viability were normalized to untreated cells. Symbols and bars represent the mean ± standard error of the mean (SEM) of at least three independent experiments. (**C**) Percentage of cells in each phase of the cell cycle in breast cancer cell lines treated with vehicle or 1.25 μM of EB-3D for the indicated time points. Data are presented as mean ± SEM of three independent experiments. (**D**) Western blots depicting changes in protein expression in breast cancer cells following treatment with vehicle or 1 μM of EB-3D for the indicated time points. Lysates were made and probed with the indicated antibodies. Quantification and statistical analysis are depicted in Figure S2A–C. (**E**) Evaluation of cell death by trypan blue exclusion assay and (**F**) Annexin V-Propidium Iodide (AV-PI) flow cytometry analysis. Breast cancer cells were treated with 1 μM of EB-3D for 72 h and then medium was replaced with fresh medium with (EB-3D continuous) or without (EB-3D washout) the ChoKα inhibitor for further 72 h. Data are represented as mean ± SEM of four independent experiments. Statistical significance was determined using ANOVA with Newman–Keuls or Bonferroni correction. Asterisks indicate a significant difference between the treated and the control group. * *p* < 0.05, ** *p* < 0.01, *** *p* < 0.001, **** *p* < 0.0001.

As previously reported, EB-3D was able to inhibit the purified ChoKα1 enzyme with an IC_50_ of 1 μM [24]. To evaluate if EB-3D-mediated ChoKα inhibition effectively reduces choline metabolites, ^1^H-NMR spectra were analyzed after treatment of the MDA-MB-231 breast cancer cell line with the ChoKα1 inhibitor. The variation of choline metabolites over time is summarized in Figure 1A.

After 24 h of treatment with 1 μM of EB-3D, the pool of free choline (Cho) was about twofold (212.5 ± 22.1%) compared to control and the gap was maintained at 48 h (180.4 ± 12.3%). On the contrary, EB-3D treatment significantly reduced the amount of the ChoKα reaction product over time, halving the phosphocholine (PCho) intracellular level in 48 h of incubation (48.9 ± 2.2%). Glycerophosphocholine (GPCho) levels were only slightly affected by ChoKα inhibition, while a general decrease in total choline (tCho) levels was observed over time, becoming significant at 48 h of treatment (72.0 ± 3.5%).

### 2.2. EB-3D Reduces Cell Proliferation in Breast Cancer Cell Lines

With the aim of evaluating the effects of EB-3D in breast cancer, we investigated its biological activity in MDA-MB-231, MDA-MB-468, and MCF-7 cell lines, where ChoKα is highly expressed (Figure S1). As depicted in Figure 1B, the drug induced a remarkable decrease of cell viability in all cell lines with a GI_50_ of 0.026 ± 0.003, 0.035 ± 0.002, and 0.037 ± 0.004 µM, respectively. Considering the strong antiproliferative effect on breast cancer cell lines studied, we evaluated the effect of EB-3D on cell cycle progression. In all treated cell lines, EB-3D induced an arrest of the cell cycle in the G1 phase along with a concomitant reduction of both the S and G2/M phases (Figure 1C). This cell cycle arrest was accompanied by a decrease in Cyclin E expression and consequent reduction of RB S780 and SMAD3 T8 phosphorylations. Decrease in T8 phopshorylation of SMAD3 restored its transcriptional activity driving the expression of the CDK inhibitor p21 [26]. Moreover, we found increased levels of the cell cycle inhibitor p21 in MDA-MB-231 and MDA-MB-468 (Figure 1D and Figure S2A–C) and of p16 and p27 in MCF-7 (Figure S3), consistent with an impairment of the G1 to S phase checkpoint.

To deeply investigate the effects of EB-3D over time, we treated breast cancer cells with EB-3D for up to 6 days and we analyzed cell proliferation by trypan blue exclusion assay. We observed a persistent arrest in cell proliferation after both continuous exposure to EB-3D or its withdrawal (Figure 1E), with the onset of significant apoptotic cell death only after prolonged time of treatment (Figure 1F). Interestingly, no significant differences between continuous exposure and washout have been detected.

### 2.3. EB-3D Induces a Senescence-Like Phenotype via AMPK-mTOR Signaling

**Figure 2:**
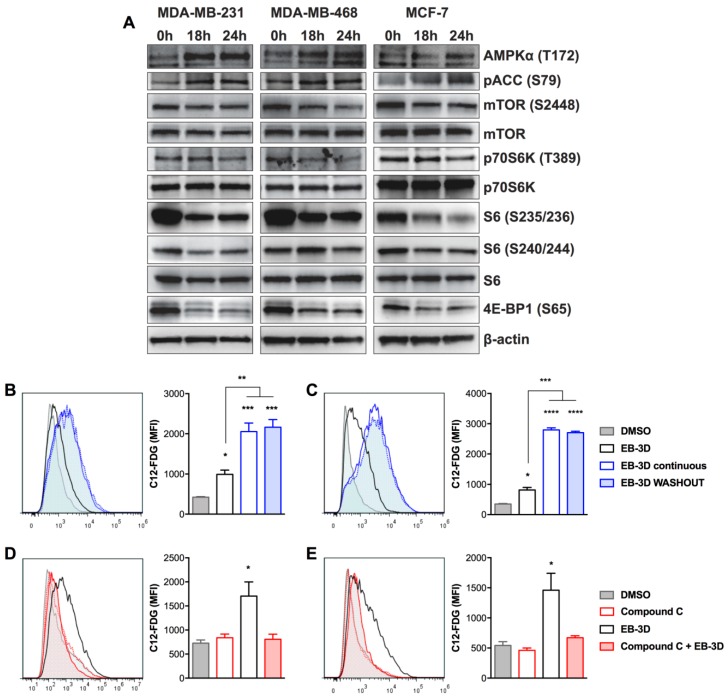
EB-3D affects AMPK-mTOR signaling pathway triggering cellular senescence. (**A**) Breast cancer cell lines were treated with 1 μM of EB-3D or vehicle for the indicated time points. Cells were then lysed and probed with the indicated antibodies. Quantification and statistical analysis are depicted in Figure S4A–C. (**B**–**E**) Flow cytometry analysis of cellular senescence using C_12_-FDG probe. MDA-MB-231 (**B**) and MDA-MB-468 (**C**) were treated with 1 μM of EB-3D for 72 h and then cells were supplied with fresh medium with (EB-3D continuous) or without (EB-3D WASHOUT) the ChoKα inhibitor for a further 72 h. MDA-MB-231 (**D**) and MDA-MB-468 (**E**) were treated with 1 μM of EB-3D for 72 h or pretreated for 2 h with 2.5 μM of Compound **C**. Data are represented as the C_12_-FDG mean fluorescence intensity (MFI) ± SEM of three independent experiments. Statistical significance was determined using ANOVA with Newman–Keuls correction. Asterisks indicate a significant difference between the treated and the control group, unless otherwise specified. * *p* < 0.05, ** *p* < 0.01, *** *p* < 0.001, **** *p* < 0.0001.

The lack of phosphocholine biosynthesis could activate energy stress and cell response to nutrient deficiencies. Indeed, we have recently demonstrated that EB-3D was able to affect the AMPK-mTOR signaling pathway in T cell acute lymphoblastic leukemia [8]. As depicted in Figure 2A and Figure S4A–C, all three cell lines—MDA-MB-231, MDA-MB-468, and MCF-7—showed a significant modulation of AMPK-mTOR pathway after EB-3D treatment. In particular, EB-3D triggered the activation of the metabolic stress sensor AMPK by the increase in T172 phosphorylation of catalytic subunit α, confirmed by the increase in S79 phosphorylation of its substrate acetyl-CoA carboxylase (ACC). On the other hand, AMPK activation inhibited the mTORC1 pathway as shown by mTOR S2448 dephosphorylation and the reduction of p70S6K (T389), 4E-BP1 (S65), and S6 ribosomal protein (S235/236 and S240/244) phosphorylations.

Considering the prolonged and irreversible effect of EB-3D on cell proliferation and the potential involvement of the AMPK-mTOR axis in this phenomenon, we wondered if this drug might induce cellular senescence in breast cancer cells. Indeed, we observed a strong increase in senescence-associated beta-galactosidase (SA-βgal) activity, a biomarker of senescent cells, already at 72 h of EB-3D treatment in all cell lines with respect to the control (Figure 2B,C and Figure S5A). The analysis of mean fluorescence intensity (MFI) indicated that the effect was time dependent with a significant gain between 72 and 144 h (992.0 ± 107.3 vs. 2053.0 ± 213.4 for MDA-MB-231 and 815.5 ± 82.3 vs. 2798.0 ± 66.5 for MDA-MB-468) and, as expected, irreversible even after EB-3D removal (2162.0 ± 191.1 and 2704.4 ± 48.2, respectively). Since activation of the AMPK-mTOR axis has been associated with senescence [27], we treated cells in the presence of Compound **C**, a well-known inhibitor of AMPK [28]. As shown in Figure 2D,E, Compound **C** dramatically reduced EB-3D-induced SA-βgal activity, pointing out AMPK involvement in the onset of the senescence process.

### 2.4. ChoKα Inhibition Synergistically Enhances Commonly Used Chemotherapeutics Effects and Sensitizes Breast Cancer Cell Lines to Cisplatin

**Figure 3:**
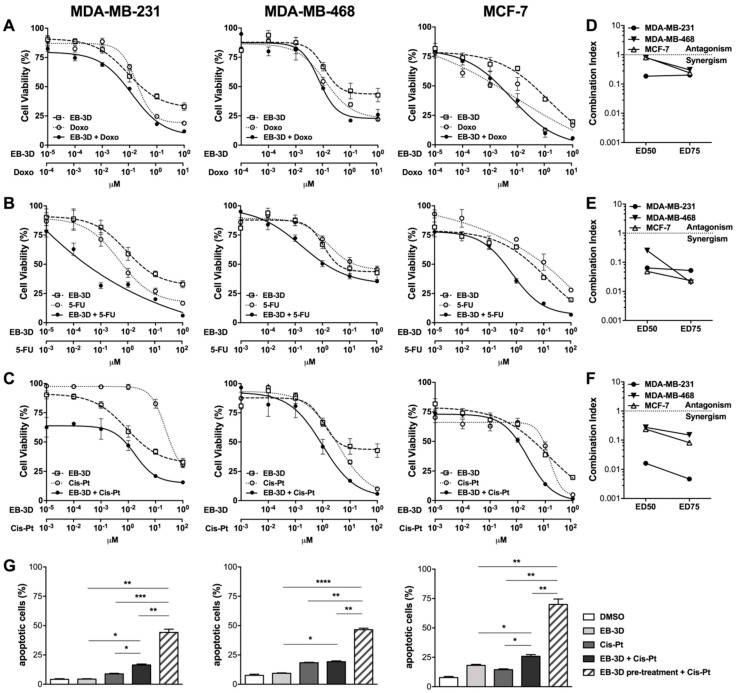
EB-3D sensitizes breast cancer cells to common treatment. (**A**–**C**) MTT cell viability assay in breast cancer cell lines treated with EB-3D in combination with doxorubicin (Doxo) (**A**), 5-fluorouracil (5-FU) (**B**), and cisplatin (Cis-Pt) (**C**) for 72 h. The percentages of cell viability were normalized to untreated cells. Symbols and bars represent the mean ± SEM of at least four independent experiments. (**D**–**F**) Combination index (CI) calculated at the ED_50_ and ED_75_ for Doxo (**D**), 5-FU (**E**), and Cis-Pt (**F**) combination, where synergism is defined by CI < 1. (**G**) Flow cytometry analysis of cell death. Breast cancer cells were treated with 1 μM of EB-3D or Cis-Pt (20 μM for MDA-MB-231 and MCF-7 or 2.5 μM for MDA-MB-468) or with the simultaneous addition of EB-3D and Cis-Pt for 72 h. Alternatively cells were pretreated with EB-3D for 72 h and then, after EB-3D removal, treated with Cis-Pt for a further 72 h. Bars represent the mean ± SEM of at least four independent experiments. Statistical significance was determined using ANOVA with Newman–Keuls correction. Asterisks indicate a significant difference between indicated groups. * *p* < 0.05, ** *p* < 0.01, *** *p* < 0.001, **** *p* < 0.0001.

We evaluated EB-3D in combination with chemotherapeutics commonly used in breast cancer treatment. All cell lines were treated for 48 h with the ChoKα inhibitor alone or in combination at a fixed molar ratio with doxorubicin (Doxo, Figure 3A), 5-fluorouracil (5-FU, Figure 3B), and cisplatin (Cis-Pt, Figure 3C). We observed that EB-3D significantly enhances cytotoxicity induced by these drugs in all cell lines (Figure 3A–C). For all tested combinations, we obtained a combination index (CI) lower than 0.3 indicating strong drug synergism (Figure 3D–F).

Given the highly synergistic effect obtained with EB-3D/Cis-Pt in MDA-MB-231 (CI(ED_75_) = 0.005), we further characterized this combination. The simultaneous addition of EB-3D and Cis-Pt significantly increased the percentage of apoptotic cells compared to single Cis-Pt treatment in cisplatin-resistant cell lines MDA-MB-231 (16.5 ± 1.4% vs. 8.8 ± 0.9%) and MCF-7 (25.8 ± 2.6% vs. 14.5 ± 1.2%), while the effect was not appreciable in the cisplatin-sensitive MDA-MB-468 (Figure 3G). More importantly, the pretreatment with EB-3D for 72 h, which induced no relevant apoptosis by itself, was able to drastically boost the apoptotic response to the subsequent treatment with Cis-Pt in all tested cell lines (44.2 ± 2.8% in MDA-MB-231, 46.6 ± 1.2% in MDA-MB-468, and 70.0 ± 4.6% in MCF-7 cells) (Figure 3G). Analogous results were obtained treating MDA-MB-231 cells with a cocktail of Doxo, 5-FU, and Cis-Pt in the presence of EB-3D. As depicted in Figure S6, EB-3D treatment at sublethal doses was able to significantly enhance the cytotoxicity of the anticancer drug cocktail.

### 2.5. Targeting ChoKα Activity Impacts Cell Migration and Invasion in the Highly Aggressive MDA-MB-231 Cells

**Figure 4:**
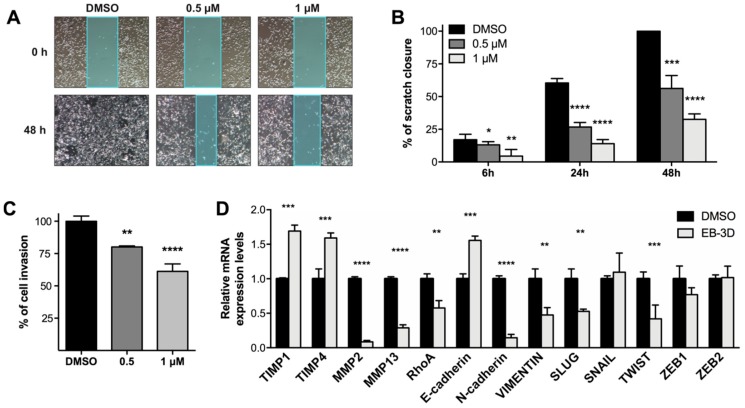
EB-3D impairs MDA-MB-231 motility and invasiveness. (**A**) Representative images of wound closure at the beginning and end of the scratch experiment, 10× magnification. (**B**) Bar graphs showing the relative quantification of the distance between scratch edges. Confluent MDA-MB-231 monolayer was scratched and treated with EB-3D at the indicated concentrations and monitored at 6, 24, and 48 h. (**C**) Relative quantification of BME-based invasion assays performed with MDA-MB-231 pretreated with EB-3D for 24 h at the indicated doses. Data are represented as mean ± SEM of four independent experiments. (**D**) Relative mRNA expression levels of EMT-related genes assessed in MDA-MB-231 cells treated with 1 μM of EB-3D by qRT-PCR. Data were normalized by the expression levels of the housekeeping gene *GUS* and expressed as a fold change relative to untreated cells (DMSO) using the 2-ΔΔCt method. Data are represented as mean ± SEM of three independent experiments. Statistical significance was determined using Student’s *t*-test or ANOVA depending on the type of data. For multiple test comparison, Newman–Keuls corrections was applied. Asterisks indicate a significant difference between the treated and the control group. * *p* < 0.05, ** *p* < 0.01, *** *p* < 0.001, **** *p* < 0.0001.

Since it has been demonstrated that inhibition of ChoKα can reduce motility and invasiveness of tumor cells [3,29], we evaluated if EB-3D was able to suppress migration and invasion in the highly metastatic MDA-MB-231 cell line. Figure 4A shows representative images of the wound-healing assay in MDA-MB-231 cells treated with different concentrations of EB-3D. The inhibition of ChoKα mediated by EB-3D resulted in a dramatic impairment of migration that was no longer able to close the scratch in a concentration-dependent manner (Figure 4B). In addition, a cell matrix invasion assay was performed in MDA-MB-231 cells pretreated with EB-3D for 24 h. ChoKα inhibition significantly impaired the ability of MDA-MB-231 to pass through a basal membrane extract (BME)-coated transwell within 24 h (Figure 4C). Considering the ability of EB-3D to reduce cell migration and invasion, we assessed the expression of epithelial-mesenchymal transition (EMT)-related genes. In particular, we found a significant reduction of EMT-associated transcription factors SLUG, TWIST, and RhoA GTP-ase. Consequently, we observed the classical pattern of E-cadherin upregulation and both N-cadherin and Vimentin downregulation. Finally, matrix metalloproteinase MMP2 and MMP13 were found downregulated in agreement with the increase of tissue inhibitors of metalloproteinase TIMP1 and TIMP4 (Figure 4D).

### 2.6. In Vivo Tumor Growth Inhibition and Senescence Induction

The antitumor potential of EB-3D was assessed in vivo using a syngeneic murine breast cancer model represented by E0771 cells, a highly aggressive mammary adenocarcinoma cell line derived from a C57BL/6 mouse. The 88% of sequence homology between human and mouse ChoKα1 and the conservation of the amino acids involved in the ChoKα-EB-3D interaction (Figure S7) suggest that the binding of our compound with murine ChoKα1 should be considered to be preserved [24]. Indeed, the antiproliferative effect of EB-3D was tested on murine E0771 cells, showing a significant reduction in cell viability with a GI_50_ = 0.31 μM, lower than the reference compounds MN58b and RSM-932A (Figure S8A). 

**Figure 5:**
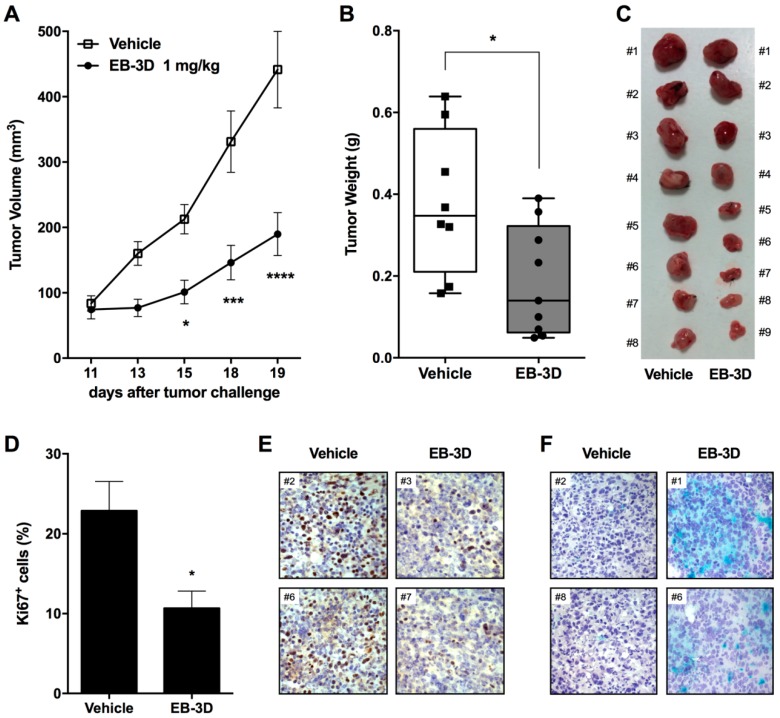
ChoKα inhibition impairs mammary tumor growth in syngeneic orthotopic E0771-C57BL/6 mouse model. (**A**) Average mammary E0771 tumor volume of mice injected with either vehicle (DMSO) or 1 mg/kg of EB-3D (*n* = 8/treatment). (**B**) Average weight and macroscopic images (**C**) of resected tumors at the conclusion of the experiment. Values are depicted as mean ± SEM. Tumors were measured in two dimensions and tumor volume V (mm^3^) was calculated according to the formula V = (D × d^2^)/2, where D and d are the major and minor perpendicular tumor diameters, respectively. Differences between control and treated mice were analyzed using Student’s *t*-test using Bonferroni correction. (**D**) Quantitative analysis of Ki67 positive cells from E0771-C57BL/6 treated or not with 1 mg/kg of EB-3D. (**E**) Representative immunohistochemical micrographs of Ki67 positive cells (brown nuclei) and (**F**) β-galactosidase (X-gal)-staining. Nuclei have been counterstained by Meyer’s Hematoxylin (Magnification 10×). Statistical significance was determined using Student’s *t*-test or ANOVA, using Bonferroni correction. Asterisks indicate a significant difference between the treated and the control group. * *p* < 0.05, *** *p* < 0.001, **** *p* < 0.0001.

As shown in Figure 5A, E0771 cells were injected orthotopically into the mammary fat pad of female mice, and after treatment with EB-3D (1 mg/kg), tumor growth was significantly impaired when compared with the control group. Notably, tumor volume reduction was of about 210% (Figure 5B,C) with respect to the vehicle-treated mice just after four administrations. Immunohistochemical analysis showed that the proliferation marker Ki67 was significantly reduced in tumors resected from mice treated with EB-3D compared to controls (Figure 5D,E). In addition, X-gal staining revealed the induction of a senescence-like phenotype even in an in vivo setting, confirming the results obtained in vitro (Figure 5F). Under a safety profile, no significant weight loss (Figure S8B), behavioral changes, and fur appearance were observed in treated mice.

### 2.7. Metastasis Formation Is Reduced by EB-3D Treatment

Since we observed that ChoKα inhibition was involved in cell migration and invasion, we evaluated EB-3D capability to reduce metastasis formation in vivo.

**Figure 6:**
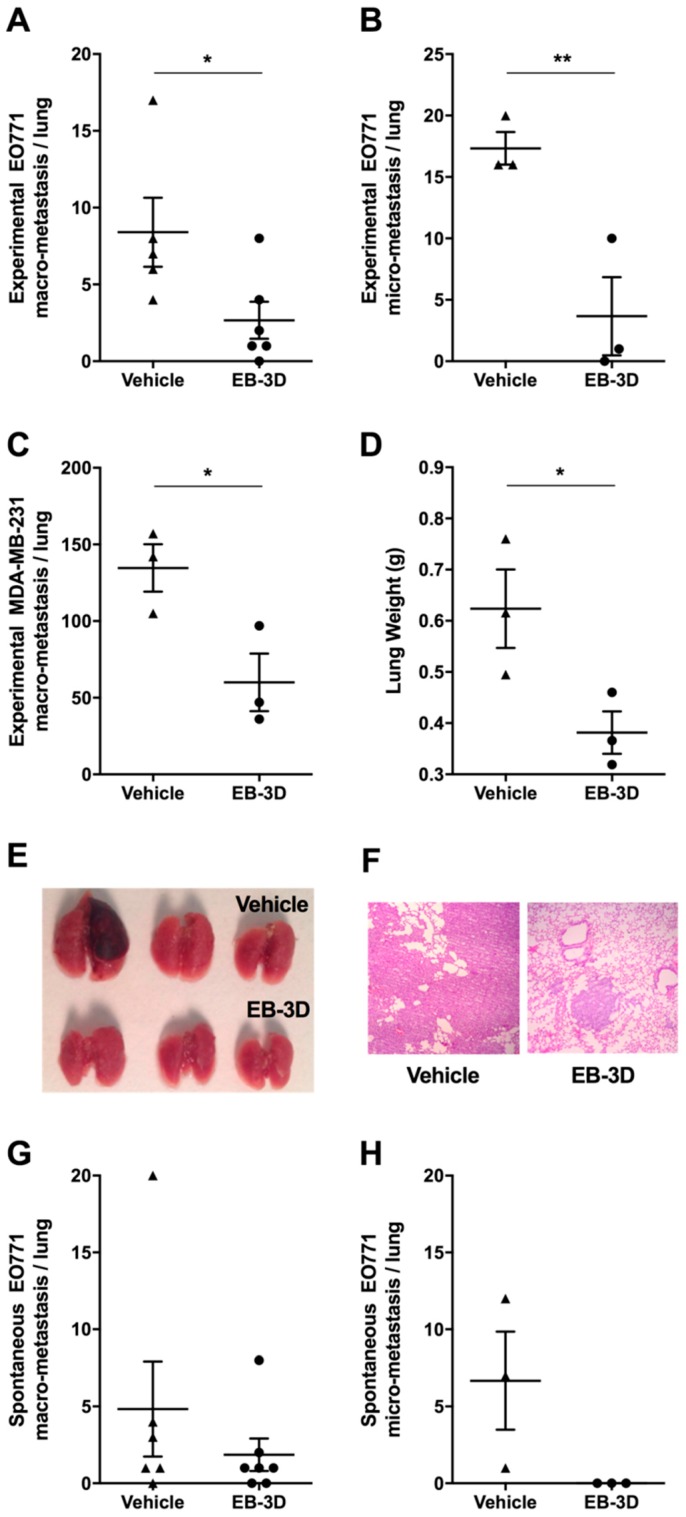
ChoKα inhibition reduces in vivo lung metastasis formation. (**A**) Number of experimentally induced lung macrometastasis and (**B**) micrometastasis after iv injection of E0771 cells and 3 weeks treatment with either vehicle (DMSO, n = 5) or 2.5 mg/kg of EB-3D (n = 6); (**C**) Number of experimentally induced lung macrometastasis after iv injection of human MDA-MB-231 cells in NOD/SCID mice and 7 weeks treatment with either vehicle (DMSO, n = 3) or 2.5 mg/kg of EB-3D (n = 3), (**D**) average lung weight, and (**E**) macroscopic images of resected lungs at the conclusion of the experiment. (**F**) Representative H&E staining performed on lung tissue sections from control and treated xenograft MDA-MB-231-NOD/SCID mice (Magnification 10×). (**G**) Number of spontaneous lung macrometastasis and (**H**) micrometastasis after E0771 primary tumor removal. E0771-C57BL/6 mice were treated intraperitoneally (ip) for 4 weeks every other day with either vehicle (DMSO, n = 5) or 2.5 mg/kg of EB-3D (n = 7). Values are depicted as mean ± SEM. Differences between control and treated mice were analyzed using Student’s *t*-test with Bonferroni correction. * *p* < 0.05, ** *p* < 0.01.

Experimental metastasis models obtained after the iv injection of murine E0771 or human MDA-MB-231 cells were treated with EB-3D at the dose of 2.5 mg/kg. As shown in Figure 6A–C, after lung removal, the number of macrometastases as well as of micrometastases was significantly reduced in treated mice when compared with control lungs. In addition, a significant reduction of resected lung weight was appreciable after EB-3D treatment (Figure 6D,E).

Hematoxylin/eosin staining of lung tissue sections showed that, unlike untreated mice where the lung anatomical architecture was completely substituted by large metastasis, EB-3D-treated samples revealed only small metastatic lesions (Figure 6F).

Finally, a model of spontaneous metastases, obtained after the removal of primary/orthotopic E0771 mammary tumors, was employed. The treatment of mice after primary tumor removal showed that, although not significant, a reduction of the number of spontaneous lung macro- and micrometastasis (Figure 6G,H). However, the observed trend was corroborated by the statistically significant reduction of experimentally induced E0771 lung macro- and micrometastasis (Figure 6A,B) in EB-3D treated mice compared to untreated ones.

## 3. Discussion

Literature studies have deemed ChoKα as a novel cancer target due to its role in survival signaling and in supporting cell proliferation. Moreover, although the oncogenic properties of ChoKα are not fully elucidated, it appears critical for the survival of cancer cells [8,18,30]. The small molecule EB-3D specifically targets the ChoKα enzyme in breast cancer cells, as pointed out by the decrease of PCho and the matched decrease of tCho levels in water-phase extracts of treated cells. We also observed a twofold increase in Cho levels after treatment that can be explained as the accumulation of ChoKα substrate, supporting the enzymatic inhibition. In different breast cancer cell lines, EB-3D induces a strong and irreversible cell proliferation arrest, with the onset of significant cell death only after prolonged time of high dose of drug exposure (20–30-fold GI_50_). This behavior has been described for other biscationic symmetrical ChoKα inhibitors [23,31], while nonsymmetrical compounds do not affect cell proliferation and viability [18,32]. 

The lack in PCho biosynthesis has been demonstrated to induce cell growth arrest and accumulation of cells in middle-to-late G1. Moreover, the increase in PCho level is a requirement for the transition through the G1 restriction point [33,34] and its reduction is consistent with the reported cell cycle arrest in the G0/G1 phase induced by EB-3D treatment. This late G1 restriction point, known also as cell growth or metabolic check-point, is dependent on mTOR signaling and cyclin E activation. Recent evidence showed that the activation of these signals is controlled by the metabolic sensor AMPK, which is able to suppress the mTOR signal in response to energy stress and nutrient-poor conditions [35]. Immunoblot data highlighted a modulation of the AMPK-mTOR metabolic pathway induced by EB-3D treatment. Indeed, Trousil et al. recently described that the symmetrical biscationic ChoKα inhibitor ICL-CCIC-0019 reduces mitochondria respiration and ATP production, leading to AMPK activation without increasing reactive oxygen species (ROS) production [23], revealing a noncanonical mitochondrial damage response [36]. We report the activation of the AMPK stress sensor that caused the reduction of mTOR phosphorylation and of its downstream targets 4E-BP1, p70S6K, and S6 upon treatment with EB-3D. The dephosphorylated form of 4E-BP1, which sequesters the eukaryotic translation initiation factor 4E (eIF4E), together with the absence of the active hyperphosphorylated form of S6, prevents the initiation and progression of the mRNA translation process. Collectively, these data suggest that ChoKα inhibition by EB-3D is sensed like a metabolic insult in breast cancer cells causing the dephosphorylation of the mTORC1 final effectors required for protein synthesis. These observations corroborate the observed reduction in cell proliferation and G0/G1 cell cycle arrest following EB-3D treatment.

The arrest of cell proliferation is maintained even after compound removal, suggesting that ChoKα inhibition is irreversible or at least that the damages caused by EB-3D are permanent, conversely to what has been reported for the nonsymmetrical ChoKα inhibitor V-11-0711 [18]. Indeed, to the best of our knowledge, we proved for the first time that cellular senescence can be induced by targeting choline metabolism in breast cancer. The pharmacological inhibition of ChoKα with EB-3D significantly increases the activity of the senescent marker SA-βgal in all tested breast cancer cells and at the same time it is important to note that this phenomenon occurs also in the in vivo E0771-C57BL/6 syngeneic model. In addition, the twofold increase of the GPCho/PCho ratio observed (Figure S1B) has also been described as a feature of senescent cells, independently from the type of senescence [37]. The induction of senescence-like phenotype seems to be cancer specific since EB-3D treatment did not induce SA-βgal activity in healthy mammary cells MCF10A and normal human fibroblasts (Figure S5D).

The involvement of AMPK signaling in triggering senescence has been already described. In this context, the increased AMP/ATP ratio and AMPK activity were observed during cellular senescence in fibroblasts [38]. Consistently, sustained AMPK activation was observed during radiation-induced senescence [39], although other research groups report that activation of AMPK prevents H_2_O_2_-induced senescence [40,41] triggering autophagy [42]. In this work, we demonstrated that the activation of AMPK by EB-3D appears to drive cellular senescence in breast cancer cells. Indeed, AMPK inhibition by Compound **C** reverted EB-3D-induced senescence-like phenotype. Of note, the induction of AMPK is a rapid event, occurring within 18 h of EB-3D treatment, and probably is not directly correlated with the reduction of pCho that does not occur before 24–48 h. Thus, it will be important to further study the possible relationship between the inhibition of ChoKα and the induction of metabolic stress. In this context, Falcon et al. demonstrated different effects on cell viability between ChoKα silencing and pharmacological inhibition by V-11-0711, suggesting a role for the ChoKα protein itself in promoting cancer cell survival that is independent of its catalytic activity [18].

Whilst cellular senescence is known to be a permanent and irreversible process, the senescence-associated secretory phenotype (SASP) has been pointed out as a potential strategy to promote tumor progression [43] and drug resistance [44]. Thus, the induction of cellular senescence could be a double-edged sword in cancer. Indeed, considering senescence as an irreversible state of growth arrest with the complete loss of proliferation potential, senescence could be perceived as a successful outcome of therapy, since the cells are reproductively “dead” [45]. Moreover, induced senescence can stimulate immunological response helping the eradication of the tumor cells [46]. We demonstrated that EB-3D-induced senescence sensitizes breast cancer cells to the apoptotic effect of cisplatin. This result reveals that the onset of cellular senescence in breast cancer cells might be advantageous. EB-3D also enhances the chemotherapeutic effects of 5-FU and doxorubicin, significantly lowering their GI_50_ values. All together, these data suggest that ChoKα inhibition could be a potential neoadjuvant and could enhance the effects of conventional chemotherapy for breast cancer, although further studies are needed.

In this work, we have also demonstrated the efficacy of EB-3D as a potent antitumor agent in vivo in a syngeneic orthotopic mouse model. It is worth noting that the effect of the drug is evident already after the second administration of 1 mg/Kg intraperitoneally (ip), indicating a potent antitumor effect and favorable pharmacokinetics. Importantly, we did not observe any sign of apparent toxicity.

Since ChoKα is the first enzyme involved in phosphatidylcholine biosynthesis, the most abundant membrane lipid, it is reasonable to assume that ChoKα plays an important role in cell membrane stability and therefore in migration and invasion processes. EB-3D-mediated ChoKα inhibition drastically reduces tumor cell motility and invasiveness in vitro in the highly aggressive MDA-MB-231. Indeed, it has been recently reported that ChoKα inhibition suppresses epithelial-to-mesenchimal (EMT) transition in glioblastoma [47]. In agreement with Koch et al., we found a deregulation of EMT-related transcription factors of the SNAIL, ZEB, and TWIST families and the canonical E-cadherin/N-cadherin switching. The high mortality rate associated with triple negative breast cancer (the most aggressive) is due primarily to the onset of metastases, mainly targeting lung, liver, bones, and brain. Hence, research efforts should focus also on the development of new therapies for prevention of secondary metastatic lesions. For these reasons, the antimetastatic effect of EB-3D was tested also in vivo using both allogeneic and xenogeneic models. Indeed, an essential component for testing new pharmacological agents is the assessment of their efficacy in preclinical settings. However, very few preclinical models that incorporate the relevant features of human metastatic disease are available. We were able to provide preliminary evidence of reduction of spontaneous lung metastatic nodules in mice treated with EB-3D after primary tumor resection, but the effect became significant when tumor cells were injected intravenously.

## 4. Materials and Methods

### 4.1. Cell Lines and Culture Conditions

Human breast adenocarcinoma MCF-7 (ATCC cat n. HTB-22; Manassas, VA, USA) were grown in low-glucose DMEM (PanBiotech, Aidenbach, Germany), MDA-MB-231, and MDA-MB-468 cell lines (provided by Dr. R. Giavazzi, Istituto M. Negri, Milan, Italy and authenticated by STR method) in DMEM (Gibco, Thermo Fisher Scientific, Waltham, MA, USA). Both media were supplemented with 10% fetal bovine serum (FBS), glutamine (2 mM), penicillin (100 U/mL), and streptomycin (100 µg/mL) (all from Thermo Fisher Scientific, Waltham, MA, USA). Mouse breast cancer E0771 cells (provided by Dr. R. Giavazzi, Istituto M. Negri, Milan, Italy and authenticated by STR method) were grown in complete DMEM medium supplemented with 20% FBS. All cell lines were cultured at 37 °C, 5% CO_2_ for no longer than 15 passages. Mycoplasma testing was periodically performed using Venor GeM OneStep Mycoplasma Detection Kit (Minerva Biolabs, Berlin, Germany). In every experiment, the DMSO concentration never exceeded 0.5%.

### 4.2. Cell Viability Assay and Drug Combination Sensitivity Assay

The cytotoxic activity of the selected drugs or drug combinations was determined after 72 h of treatment by MTT colorimetric assay (Sigma-Aldrich, Milan, Italy). In drug combination assays, synergism was determined by the calculation of the combination index (CI) using CalcuSyn software (version 2.0, Biosoft) based on the algorithm described by Chou [48], where synergism is defined by CI < 1, additivity as CI = 1, and antagonism as CI > 1. In additional experiments, the accurate determination of the cell proliferation rate was determined by trypan blue exclusion assay. Cells in exponential growth were treated with EB-3D (time 0) and then collected at the indicated time points. Cells were resuspended in 0.4% trypan blue solution (Thermo Fisher Scientific) and counted on a hemocytometer. Only trypan blue negative cells were considered viable cells. Detailed protocols are available in the Supplementary Materials and Methods.

### 4.3. Cell Cycle Distribution Analysis

For flow cytometric analysis of DNA content, 5 × 10^5^ cells in exponential growth were treated with different concentrations of EB-3D for 24 h. Cells were then collected, centrifuged, and fixed with ice-cold ethanol (70%). The cells were then treated with a buffer containing RNAse A (Qiagen, Hilden, Germany) and 0.1% Triton X-100 (Sigma-Aldrich) and then stained with propidium iodide (PI) (Sigma-Aldrich). Samples were analyzed on a Cytomics FC500 flow cytometer (Beckman Coulter, Brea, CA, USA). DNA histograms were analyzed using MultiCycle for Windows (Phoenix Flow Systems).

### 4.4. Magnetic Resonance Spectroscopy (^1^H-MRS)

MDA-MB-321 breast cancer cells were seeded and cultured for 24 h in complete growth medium and then treated with EB-3D or DMSO for the indicated time points. Water-soluble extracts were obtained using the dual-phase extraction method. The detailed protocol is in the Supplementary Materials and Methods.

### 4.5. Annexin-V/PI Assay

Surface exposure of phosphatidylserine on apoptotic cells was measured by flow cytometry with Cytomics FC500 (Beckman Coulter) by simultaneously adding annexin-V (AV) conjugated to fluorescein isothiocyanate (FITC) and propidium iodide (PI) to cells according to the manufacturer’s instructions (Annexin-V-Fluos staining kit, Roche Diagnostic, Indianapolis, IN, USA).

### 4.6. C_12_-FDG Senescence Assay

For flow cytometric analysis of cellular senescence, cells were treated with EB-3D for 72 h and then the medium was replaced with fresh medium containing EB-3D or DMSO for the next 72 h. For rescue experiments, cells were pretreated with 2.5 μM of Compound **C** (Santa Cruz Biotechnology, Dallas, TX, USA) for 2 h and then treated with EB-3D for the next 3 days. Senescence was evaluated by flow cytometry on a Cytomics FC500 flow cytometer (Beckman Coulter) using di-β-D-galactopyranoside (C_12_-FDG) (Invitrogen, Thermo Fisher Scientific, Waltham, MA, USA), a fluorogenic substrate for β-gal activity, as previously described [49].

### 4.7. Western Blot Analysis

Total cellular proteins were extracted from cells using T-PER lysis buffer (Pierce, Milano, Italy) containing phosphatase and protease inhibitors. The protein concentration was determined using the BCA protein assay reagents (Thermo Scientific). Equal amounts of protein were resolved using SDS-PAGE and transferred to PVDF Immobilon-P Membrane (Merck Millipore, Darmstadt, Germany). Membranes were saturated with BSA 3% and probed overnight at 4 °C with specific primary antibodies (listed in the Supplementary Materials and Methods). Membranes were then washed and incubated with HRP-labeled secondary antibodies (goat anti-rabbit or anti-mouse IgG; Perkin Elmer, Waltham, MA, USA). All membranes were stained using ECL Select (GE Healthcare, Catania, Italy) and visualized with Alliance 9.7 (UVITEC, Cambridge, UK). To ensure equal protein loading, each membrane was reprobed with β-actin antibody (Sigma-Aldrich).

### 4.8. Scratch-Migration Assay

Nearly confluent MDA-MB-231 cells were gently wounded through the horizontal and vertical axis using a pipette tip. Cells were washed twice to remove cell debris and then treated with EB-3D at the indicated concentration for 48 h. Each time point image was captured at 7× magnification under a stereomicroscope. The distance between the two edges of the scratch was quantified using Adobe Photoshop CS6.

### 4.9. Cultrex BME Cell Invasion Assay

MDA-MB-231 cells were added to 24-well Transwell inserts of the Cultrex BME (Basal Membrane Extract) Cell Invasion Assay (Trevigen, Gaithersburg, MD, USA) according to the manufacturer’s manual. Invasion was measured 24 h after plating at 485–520 nm using the VICTOR microplate reader (Perkin Elmer).

### 4.10. Quantitative Real-Time PCR (qRT-PCR)

Total RNA was extracted with TRIzol reagent (Invitrogen) according to the manufacturer’s protocol and RNA purity and concentration were determined by measuring the spectrophotometric absorption at 260 and 280 nm on NanoDrop ND-1000. One microgram of RNA was reverse-transcribed into first strand cDNA using Superscript III Reverse Transcriptase (Life technologies, Paisley, UK) and random primers following the manufacturer’s instructions. Quantitative real-time PCR (qRT-PCR) reaction was carried with SYBR Green PCR Master Mix (Life Technologies) with ABI 7900 system (Applied Biosystems, Foster City, CA, USA) using specific primers listed in the Supplementary Materials and Methods. Each reaction was performed in triplicate and mRNA levels of target genes were normalized by the housekeeping gene *GUS* and expressed as a fold change relative to control using the 2^−ΔΔ*C*t^ method. Data are represented as mean ± standard error of the mean (SEM) of three independent experiments.

### 4.11. In Vivo Tumor Growth

Animal experiments were approved by the local animal ethics committee (OPBA, Organismo Preposto al Benessere degli Animali, Università degli Studi di Brescia, Italy) and were performed in accordance with national guidelines and regulations. Procedures involving animals and their care conformed with institutional guidelines that comply with national and international laws and policies (EEC Council Directive 86/609, OJ L 358, 12 December 1987) and with “ARRIVE” guidelines (Animals in Research Reporting In Vivo Experiments).

Seven-week-old C57BL/6 female mice were orthotopically injected into the mammary fat pad with 4 × 10^5^ E0771 mammary carcinoma cells. When tumors were palpable, mice were randomized to control and treated groups. Treatment was performed every other day by intraperitoneal (ip) injection of EB-3D (1 mg/kg) or vehicle (DMSO) in 100 µL final volume. Tumors were measured in vivo using a calipers:tumor volume V (mm^3^) that was calculated according to the formula V = (D × d^2^)/2, where D and d are the major and minor perpendicular tumor diameters in mm, respectively.

Tumor volume data were analyzed with a two-way analysis of variance, and individual group comparisons were evaluated by the Bonferroni correction.

At the end of the experimental procedure, tumors were harvested, weighted, photographed, and embedded in OCT and frozen for histological processing.

### 4.12. In Vivo Metastasis Models

Animal experiments were approved by the local animal ethics committee (OPBA, Organismo Preposto al Benessere degli Animali, Università degli Studi di Brescia, Italy) and were executed in accordance with national guidelines and regulations. Procedures involving animals and their care were conformed with institutional guidelines that comply with national and international laws and policies (EEC Council Directive 86/609, OJ L 358, 12 December 1987) and with “ARRIVE” guidelines (Animals in Research Reporting In Vivo Experiments).

For spontaneous metastases, 5 × 10^5^ E0771 cells were injected into the mammary fat pad of C57BL/6 female mice and tumors were resected when they reached the 8 × 13 mm size. After one week of recovery, mice were treated ip every other day for four weeks with vehicle or EB-3D (2.5 mg/kg) in 100 µL final volume.

For experimentally induced metastasis, murine E0771 (2 × 10^5^) or human MDA-MB-231 (8 × 10^5^) breast cancer cells in 100 µL of PBS were injected intravenously into the tail vein of 7-week-old C57BL/6 or NOD/SCID female mice, respectively. Animals were treated every other day by ip injection with EB-3D (2.5 mg/kg) or vehicle (DMSO) in 100 µL final volume for 3 (for E0771 cells) or 7 weeks (for MDA-MB-231 cells).

At the end of the experimental procedures, mice were sacrificed, lungs were harvested, weighted, formalin-fixed, and macrometastases were counted under a dissecting microscope. Lungs were then embedded in paraffin for histological processing and for the counting of micrometastases. Hematoxylin and eosin (H&E) staining was performed on five sections/specimens and lesions containing 50–200 cells were considered as micrometastases.

### 4.13. Histological Analyses

OCT-embedded E0771 tumors were sectioned and stained with anti-Ki67 antibody or with x-gal using Senescence Cells Histochemical Staining Kit (Sigma-Aldrich) according to the manufacturer’s instructions. Nuclei were counterstained by Meyer’s Hematoxylin.

Formalin-fixed, paraffin-embedded samples were sectioned, dewaxed, hydrated, and stained by H&E. All images were captured by a video-confocal microscope (T1 microscope, Zeiss, Oberkochen, Germany) using a 10× objective. Images were compiled for figures using Adobe Illustrator (Adobe Systems Inc., San Jose, CA, USA).

### 4.14. Statistical Analyses

Graphs and statistical analyses were performed using GraphPad Prism software (GraphPad, La Jolla, CA, USA). All data in graphs represented the mean of at least three independent experiments ± SEM. Statistical significance was determined using Student’s *t*-test or ANOVA (one- or two-way) depending on the type of data. For multiple test comparison, Bonferroni or Newman–Keuls corrections were applied. Asterisks indicate a significant difference between the treated and the control group, unless otherwise specified. * *p* < 0.05, ** *p* < 0.01, *** *p* < 0.001, **** *p* < 0.0001.

## 5. Conclusion 

In conclusion, the new ChoKα inhibitor EB-3D provided excellent antiproliferative effects in vitro and proved to be an effective antitumoral agent in a preclinical aggressive breast cancer model. For these reasons, we claim that EB-3D is worthy of further studies in breast cancer as well as in other tumors.

## Supplementary Materials

The following are available online at http://www.mdpi.com/2072-6694/10/10/391/s1, Figure S1: Immunoblot analysis of ChoKα protein level in human breast cancer cell lines; Figure S2: Densitometric analysis of Western blot represented in Figure 1D; Figure S3: Time-course immunoblot analysis of cell cycle inhibitors in MCF-7 cells; Figure S4: Densitometric analysis of Western blot represented in Figure 2A; Figure S5: Additional analysis of senescence-like phenotype in normal and cancer cell lines; Figure S6: Flow cytometry analysis of cell death induced by combination treatment in MDA-MB-231 cells; Figure S7: ChoKα protein sequence alignment generated by EMBOSS Needle Global alignment tools between human and mouse protein sequences; Figure S8: Validation of EB-3D effects and toxicity in breast cancer mice model; Supplementary Materials and Methods.
