# Supplementary material for: Choline Kinase Alpha Inhibition by EB-3D Triggers Cellular Senescence, Reduces Tumor Growth and Metastatic Dissemination in Breast Cancer
Source: Cancers (Basel). 2018 Oct 22;10(10):391. doi: 10.3390/cancers10100391 (PMC6209942; doi:10.3390/cancers10100391)
Supplement: Supplementary file 1 [file cancers-10-00391-s001.pdf]

Supplementary material

Choline Kinase Alpha Inhibition by EB-3D Triggers Cellular Senescence, Reduces Tumor Growth and Metastatic Dissemination in Breast Cancer

Elena Mariotto, Giampietro Viola, Roberto Ronca, Luca Persano, Sanja Aveic, Zaver M. Bhujwalla, Noriko Mori, Benedetta Accordi, Valentina Serafin, Luisa Carlota López-Cara and Roberta Bortolozzi

Supplementary Figures

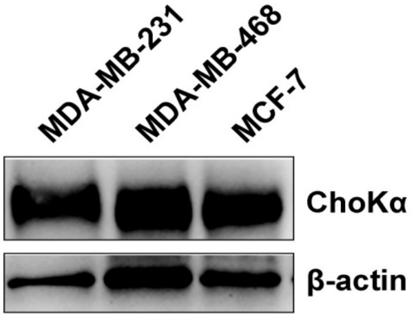

Figure S1. Immunoblot analysis of ChoKα protein level in human breast cancer cell lines.

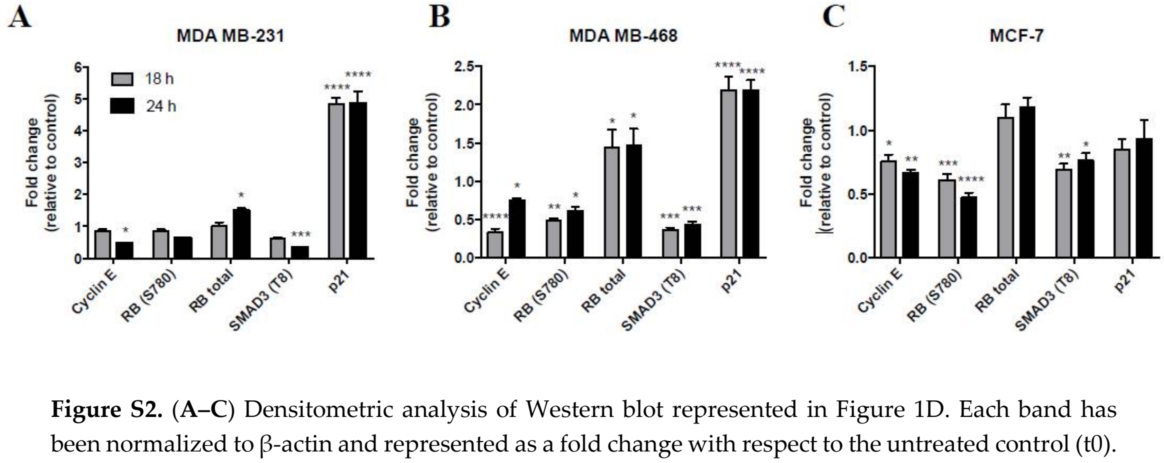

Figure S2. (**A–C**) Densitometric analysis of Western blot represented in Figure 1D. Each band has been normalized to β-actin and represented as a fold change with respect to the untreated control (t0). Data are presented as mean ± SEM of at least three independent experiments. Differences between treatment and its control were analyzed using one-way ANOVA with Bonferroni correction. * $p < 0.05$, ** $p < 0.01$, *** $p < 0.001$, **** $p < 0.0001$.

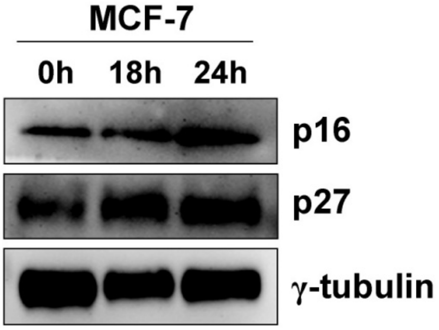

Figure S3. Time-course immunoblot analysis of cell cycle inhibitors in MCF-7 cells. MCF-7 cells were treated with 1 μM of EB-3D up to 24 h.

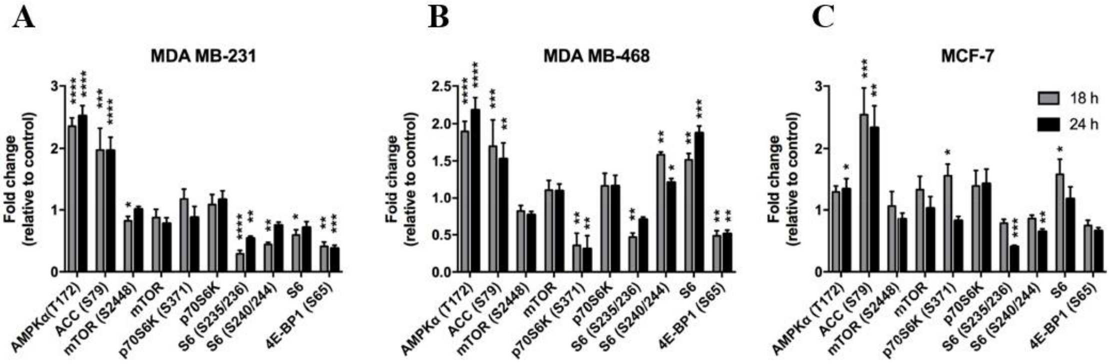

Figure S4. (**A–C**) Densitometric analysis of Western blot represented in Figure 2A. Each band has been normalized to β-actin and represented as a fold change with respect to the untreated control (t0). Data are presented as mean ± SEM of at least three independent experiments. Differences between treatment and its control were analyzed using one-way ANOVA with Bonferroni correction. * $p < 0.05$, ** $p < 0.01$, *** $p < 0.001$, **** $p < 0.0001$.

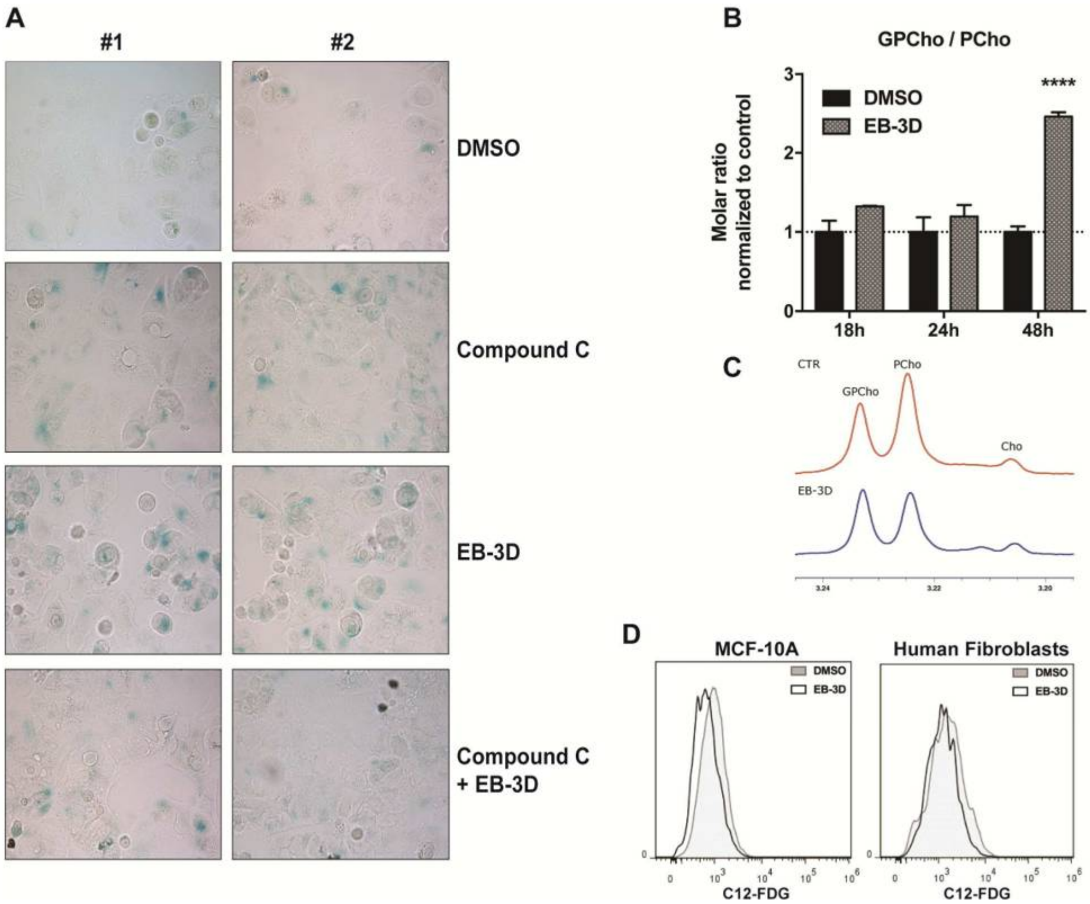

Figure S5. Additional analysis of senescence-like phenotype in normal and cancer cell lines. (**A**) β-galactosidase (X-gal)-staining in MCF-7 breast cancer cells. MCF-7 were seeded onto a chamber slide, treated with 1 μM of EB-3D or 2.5 μM of Compound C (AMPK inhibitor) for 72 h or alternatively pretreated with Compound C for 2 h, and then exposed to EB-3D for 72 h. After treatment cells, were stained according to the manufacturer's protocol. Original magnification 10×. (**B**) GPCho/PCho molar ratio quantified from ^{1}H-NMR spectra of water-soluble extracts from MDAMB-231 cells treated with DMSO or EB-3D 1 μM for the indicated time points, retrieved from Figure 1A. Metabolite levels are normalized to each time point control. Differences between treatment and its control were analyzed using one-way ANOVA with Bonferroni correction. **** $p < 0.0001$. (**C**) Representative ^{1}H-NMR spectra from the 3.20–3.25 ppm region of water-soluble extracts from MDA-MB-231 cells after 48 h of treatment with DMSO (red line) and 1 μM of EB-3D (blue line). (**D**) Flow cytometry analysis of cellular senescence using C12-FDG probe in normal mammary MCF-10A cell line and in normal human fibroblast treated with 1 μM of EB-3D for 72 h.

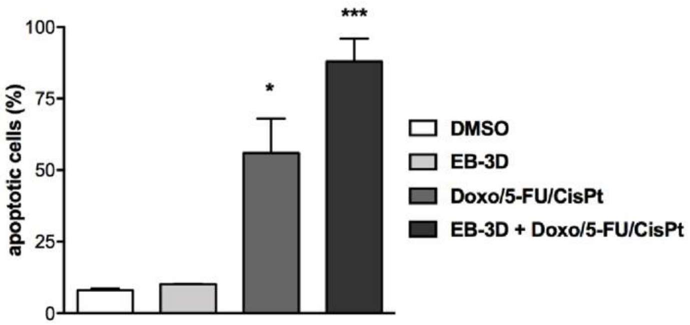

Figure S6. Flow cytometry analysis of cell death. MDA-MB-231 cells were treated with 1 μM of EB-3D or an anticancer drug cocktail (composed by Cis-Pt 20μM, 5-FU 1μM, Doxo 0.5μM) or with the simultaneous addition of EB-3D and the drug cocktail for 72 h. Bars represent the mean ± SEM of three independent experiments. Statistical significance was determined using ANOVA with Newman–Keuls correction. Asterisks indicate a significant difference between treated and control (DMSO). * $p < 0.05$, *** $p < 0.001$.

```
CHKA HUMAN         1 MKTKFCTGGEAEPSPLGLLLSCGSGSAAPAPGVGQQRDAASDLESKQLGG     50
                     ||||||||||||||||||||||| |:|||.||||||||||.:||||||||
CHKA MOUSE         1 MKTKFCTGGEAEPSPLGLLLSCG-GNAAPTPGVGQQRDAAGELESKQLGG     49

CHKA HUMAN        51 QQPPLALPPPPPLPLPLPLPQPPPPQPP-ADEQPEPRTRRRAYLWCKEFL     99
                     :..|||||||||.||||    ||||.|| |||||||||||||||||||||
CHKA MOUSE        50 RTQPLALPPPPPPPLPL----PPPPSPPLADEQPEPRTRRRAYLWCKEFL     95

CHKA HUMAN       100 PGAWRGLREDEFHISVIRGGLSNMLFQCSLPDTTATLGDEPRKVLLRLYG    149
                     ||||||||||:|||||||||||||||||||||:.|::|||||||||||||
CHKA MOUSE        96 PGAWRGLREDQFHISVIRGGLSNMLFQCSLPDSIASVGDEPRKVLLRLYG    145

CHKA HUMAN       150 AILQMRSCNKEGSEQAQKENEFQGAEAMVLESVMFAILAERSLGPKLYGI    199
                     |||:|||||||||||||.|||||||||||||||||||||||||||||:||
CHKA MOUSE       146 AILKMRSCNKEGSEQAQNENEFQGAEAMVLESVMFAILAERSLGPKLFGI    195

CHKA HUMAN       200 FPQGRLEQFIPSRRLDTEELSLPDISAEIAEKMATFHGMKMPFNKEPKWL    249
                     ||||||||||||||||||||.|||||||||||||||||||||||||||||
CHKA MOUSE       196 FPQGRLEQFIPSRRLDTEELRLPDISAEIAEKMATFHGMKMPFNKEPKWL    245

CHKA HUMAN       250 FGTMEKYLKEVLRIKFTEESRIKKLHKLLSYNLPLELENLRSLLESTPSP    299
                     ||||||||.:|||:||:.|:|:::|||:||||||||||||||||:.|.||
CHKA MOUSE       246 FGTMEKYLNQVLRLKFSREARVQQLHKILSYNLPLELENLRSLLQYTRSP    295

CHKA HUMAN       300 VVFCHNDCQEGNILLLEGRENSEKQKLMLIDFEYSSYNYRGFDIGNHFCE    349
                     ||||||||||||||||||:||||::|||||||||||||||||||||||||
CHKA MOUSE       296 VVFCHNDCQEGNILLLEGQENSERRKLMLIDFEYSSYNYRGFDIGNHFCE    345

CHKA HUMAN       350 WMYDYSYEKYPFFRANIRKYPTKKQQLHFISSYLPAFQNDFENLSTEEKS    399
                     |||||:|||||||||||:|||::|||||||||||..||||||:||:||:.
CHKA MOUSE       346 WMYDYTYEKYPFFRANIQKYPSRKQQLHFISSYLTTFQNDFESLSSEEQF    395

CHKA HUMAN       400 IIKEEMLLEVNRFALASHFLWGLWSIVQAKISSIEFGYMDYAQARFDAYF    449
                     ..||:||||||||||||||||||||||||||||||||||:||||||:|||
CHKA MOUSE       396 ATKEDMLLEVNRFALASHFLWGLWSIVQAKISSIEFGYMEYAQARFEAYF    445

CHKA HUMAN       450 HQKRKLGV    457
                     .|||||||
CHKA_MOUSE       446 DQKRKLGV    453


Identity: 402/457 (88.0%)
Similarity: 433/457 (94.7%)
Gaps: 6/457 (1.3%)
```

Figure S7. ChoKα protein sequence alignment generated by EMBOSS Needle Global alignment tools between human and mouse protein sequences (http://www.ebi.ac.uk/Tools/psa/emboss_needle/). Residues highlighted in yellow (Tyr333, Tyr345, Phe361, Trp420, Trp423, Ile433, Phe435, Tyr440) shown to be important for interaction with EB-3D or its stabilization are perfectly conserved. Also, Asp306 catalytic base for ATP hydrolysis is conserved. Vertical bar (|) indicates fully conserved residue. Colon (:) indicates conservation between groups of strongly similar properties. Period (.) indicates conservation between groups of weakly similar properties.

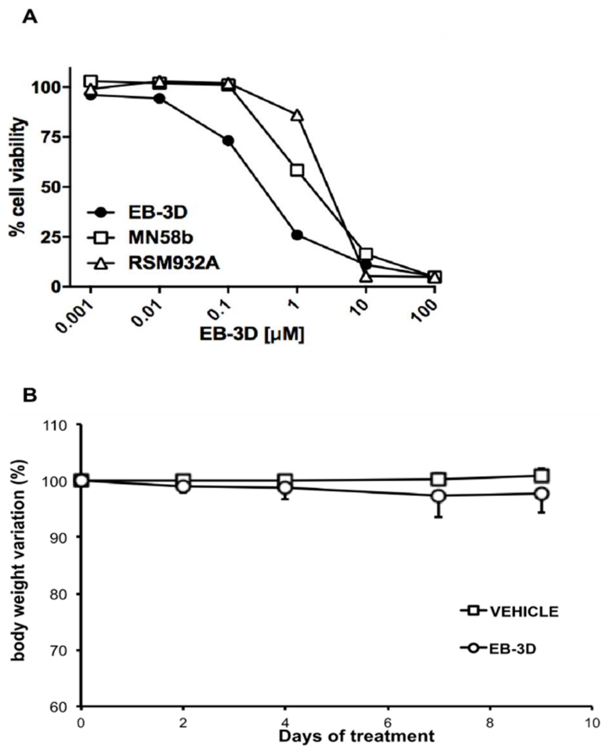

Figure S8. Validation of EB-3D effects and toxicity in breast cancer mice model. (**A**) MTT cell proliferation assay in murine EO771 breast cancer cell line treated with 1:10 serial dilution of EB-3D or the two reference compounds Mn58b and RSM-932A for 72 h. The percentages of cell viability were normalized to untreated cells. Symbols and bars represent the mean ± SEM of at least three independent experiments. (**B**) Body weight variation expressed as percentage of healthy C57BL/6 mice treated with 2.5 mg/kg compared to control group (VEHICLE). Symbols and bars represent the mean ± SEM of n = 3 mice per group.

Supplementary Materials and Methods

1. Cell Viability Assay and Drug Combination Sensitivity Assay

Individual wells of 96-well tissue-culture microtiter plates were inoculated with 100 μL of complete medium containing MDA-MB-231 or MDA-MB-468 (3.5×10^3 cells) or MCF-7 (5×10^3 cells). The plates were incubated at 37 °C in a humidified 5% CO_2 incubator for 18 h prior to the experiments to ensure exponential growth. After medium removal, 100 μL of fresh medium containing serial dilutions of single drugs or drug combinations (at a fixed molar ratio) was added to each well and incubated at 37 °C for 48/72 h. The percentage of DMSO in the medium in no case exceeded 0.5%. Cell viability was assayed by the (3-(4,5-dimethylthiazol-2-yl)-2,5diphenyl tetrazolium bromide (MTT) test. Briefly, MTT was added to each well at a final concentration of 0.5 mg/mL and incubated for 3 h at 37 °C. The quantity of formazan (presumably directly proportional to the number of viable cells) was measured by recording changes. Viable cells with active metabolism converted MTT into a purple-colored formazan product, which was next solubilized with acidified isopropanol to measure the changes in absorbance at 570 nm using a plate reading spectrophotometer (Victor, Perkin Elmer). The GI_{50} was defined as the compound concentration required to inhibit cell proliferation by 50% in comparison with cells treated with the maximum amount of DMSO (since EB-3D is solubilized in DMSO) and considered as 100% viability. Each drug concentration/combination was performed in triplicate. To determine the synergistic, additive, or antagonistic effects of the drug combinations, CalcuSyn software (version 2.0, Biosoft) based on the method of the combination index (CI) described by Chou was used (22), where synergism is defined as CI < 1, additivity as CI = 1, and antagonism as CI > 1.

In additional experiments, the accurate determination of the cell proliferation rate was determined by trypan blue exclusion assay. 7.5×10^4 cells were seeded in six-well plates and incubated at 37 °C in a humidified 5% CO_2 incubator for 18 h prior to the experiments to ensure exponential growth. Cells were then treated with EB-3D or DMSO (time 0) and then collected by trypsinization after 72 h. Alternatively, after 72 h of EB-3D treatment, cells were washed extensively and then treated with EB-3D (EB-3D continuous) or DMSO (EB-3D WASHOUT) for a further 72 h. Collected cells were resuspended in 0.4% trypan blue solution (Thermo Fisher Scientific) and counted on a hemocytometer. Only trypan blue negative cells were considered viable cells.

2. Magnetic Resonance Spectroscopy (^{1}H-MRS)

MDA-MB-321 breast cancer cells were seeded and cultured for 24 h in complete growth medium and then treated with EB-3D or DMSO for the indicated time points. Water-soluble extracts were obtained using the dual-phase extraction method. Briefly, cells were resuspended with ice-cold methanol and vigorously vortexed. Samples were incubated on ice for 15 min, mixed with chloroform (1:1), vortexed vigorously, and kept on ice for 10 min. Finally, water was added and shaken well. Samples were stored at 4 °C overnight for phase separation and later centrifuged at 15,000× *g* at 4 °C for 30 min. The upper water/methanol phase containing water-soluble cellular metabolites (Cho, PCho, and GPCho) was treated with 50 mg of chelex beads (Sigma-Aldrich, Milan, Italy) and then removed by filtration. Following methanol evaporation, the remaining water phase was lyophilized. Water-soluble extracts were resuspended in deuterated water containing 7.006×10^{-8} moles of 3-(trimethylsilyl)-propionic-2,2,3,3-d_4 acid (TSP, Sigma-Aldrich) as an internal standard for NMR spectral analysis.

NMR spectra were analyzed using Bruker Topspin software (Bruker Biospin). Signal integrals of $N(CH_3)_3$ of Cho at about 3.202 ppm, PCho at about 3.224 ppm, and GPCho at about 3.232 ppm in water-soluble extracts were determined, normalized to cell number and cell volume, and compared with the standards. To determine concentrations of water-soluble extracts, peak integrations (I_{met}) from ^{1}H spectra for PCho, GPC, and Cho were compared with that of the internal standard TSP (I_{TSP}).

3. Western Blot Analysis

Total protein extracts were isolated in T-PER lysis buffer (Pierce, Milano, Italy) containing NaCl 300 mM, orthovanadate 1 mM (Sigma, Milano, Italy), PEFABLOC SC 2 mM (Roche Biochemicals, Milano, Italy), Aprotinin 1 μg/mL (Sigma–Aldrich, Milano, Italy), Pepstatin-A 5 μg/mL (Sigma–Aldrich, Milano, Italy), and Leupeptin 1 μg/mL (Sigma–Aldrich, Milano, Italy). The protein concentration in the supernatant was determined using the BCA protein assay (Pierce, Milano, Italy). Equal amounts of protein (10 μg) were resolved using Criterion TGX precast gels (BioRad, Italy) and transferred to Immobilon-P membrane (Millipore). Membranes were blocked with 3% BSA solution for 3 h. Membranes were incubated overnight at 4 °C with primary antibodies: 4EBP1 (S65), AMPKα (T712), mTOR (S2448), p21, p70S6K (T389), S6 (S235/S236), and S6 (S240/S244) (Cell Signalling Technology, Danvers, MA); mTOR, p70S6K, S6, and ChoKα (Santa Cruz Biotechnology, Dallas, TX); SMAD3 (T8) (Biorbyt, Cambridge, UK); ACC (S79) (Millipore, Burlington, MA); Cyclin E, pRB (S78) and RB (BD Bioscience, San Jose, CA). Membranes were then washed and incubated with HRP-labeled secondary antibodies (goat anti-rabbit or anti-mouse IgG; Perkin Elmer, Waltham, MA) for 60 min at room temperature. All membranes were stained using ECL Select (GE Healthcare, Catania, Italy) and visualized with Alliance 9.7 (UVITEC, Cambridge, UK). To ensure equal protein loading, each membrane was reprobed with β-actin antibody (Sigma-Aldrich S.r.l., Milan, Italy). Western blots were quantified by densitometric analysis using ImageJ software.

4. Quantitative Real-Time PCR (qRT-PCR)

Total RNA was extracted with TRIzol reagent (Invitrogen) according to the manufacturer's protocol and RNA purity and concentration were determined by measuring the spectrophotometric absorption at 260 and 280 nm on NanoDrop ND-1000. One microgram of total RNA was reverse-transcribed into first strand cDNA using Superscript III Reverse Transcriptase (Life technologies) and random primers following the manufacturer's instructions. Quantitative real-time PCR reaction was carried with SYBR Green PCR Master Mix (Life Technologies) with ABI 7900 system (Applied Biosystems) using the following specific primers:

GUS-(F) 5′-GAAAATATGTGGTTGGAGAGC-3′
GUS-(R) 5′-GAAAATATGTGGTTGGAGAGC-3′
TIMP1-(F) 5′-GTGGCACTCATTGCTTGTGG-3′
TIMP1-(R) 5′-CAAGGTGACGGGACTGGAAG-3′
TIMP4-(F) 5′-TGCAACTACATCGAGCCCTG-3′
TIMP4-(R) 5′-TTAGGGGCCGAGATGGTACA-3′
MMP2-(F) 5′-GATCTACTCAGCCAGCACCC-3′
MMP2-(R) 5′-ACGACGGCATCCAGGTTATC-3′
MMP13-(F) 5′-ATGCAGCAAGCTCCATGACT-3′
MMP13-(R) 5′- ATCAGGAACCCCGCATCTTG-3′
RhoA-(F) 5′-GAGCCGGTGAAACCTGAAGA-3′
RhoA-(R) 5′-TTCCCACGTCTAGCTTGCAG-3′
E-cadherin-(F) 5′-CAGCCTGTCGAAGCAGGATT-3′
E-cadherin-(R) 5′-CGCTCTCCTCCGAAGAAACA-3′
N-cadherin-(F) 5′-ATTGGACCATCACTCGGCTT-3′
N-cadherin-(R) 5′-CACACTGGCAAACCTTCACG-3′
VIMENTIN-(F) 5′-GGACCAGCTAACCAACGACA-3′
VIMENTIN-(R) 5′-AAGGTCAAGACGTGCCAGAG-3′
SLUG-(F) 5′-CATCTTTGGGGCGAGTGAGT-3′
SLUG-(R) 5′-ATGGCATGGGGGTCTGAAAG-3′
SNAIL-(F) 5′-CCTCAAGATGCACATCCGAAGC-3′
SNAIL-(R) 5′-GGTTGGAGCGGTCAGCGAAG-3′
TWIST-(F) 5′-CATGTCCGCGTCCCACTAGC-3′
TWIST-(R) 5′-CCCCACGCCCTGTTTCTTTGA-3′
ZEB1-(F) 5′-GCCAATAAGCAAACGATTCTG-3′
ZEB1-(R) 5′-TTTGGCTGGATCACTTTCAAG-3′

ZEB2-(F) 5′-CGGTGCAAGAGGCGCAAACA-3′
ZEB2-(R) 5′-GGAGGACTCATGGTTGGGCA-3′

Each reaction was performed in triplicate and mRNA levels of target genes were normalized by the housekeeping gene *GUS* and expressed as a fold change relative to control using the $2^{-\Delta\Delta Ct}$ method. Data are represented as mean ± SEM of three independent experiments. Difference between DMSO and EB-3D treated cells were analyzed using ANOVA with Bonferroni correction.
